# Avian Influenza Viruses in Water Birds, Africa^1^

**DOI:** 10.3201/eid1304.061011

**Published:** 2007-04

**Authors:** Nicolas Gaidet, Tim Dodman, Alexandre Caron, Gilles Balança, Stephanie Desvaux, Flavie Goutard, Giovanni Cattoli, François Lamarque, Ward Hagemeijer, François Monicat

**Affiliations:** * Centre de Cooperation Internationale en Recherche Agronomique pour le Développement , Montpellier, France; † Wetlands International, Wageningen, the Netherlands; ‡ Viale dell’Università, Legnaro, Italy; § Office National de la Chasse et de la Faune Sauvage, Paris France

**Keywords:** avian influenza, water birds, Africa, dispatch

## Abstract

We report the first large-scale surveillance of avian influenza viruses in water birds conducted in Africa. This study shows evidence of avian influenza viruses in wild birds, both Eurasian and Afro-tropical species, in several major wetlands of Africa.

Wild water birds are considered to be the major natural reservoir for avian influenza viruses (AIV) (*1*). Large numbers of Eurasian breeding water birds overwinter in the sub-Saharan region of the African continent (*2*), where the survival of AIV is considered to be restricted by the tropical environment (*3*). Although the first reported isolation of AIV from wild birds (A/Tern/S.A./61 [H5N3]) was in Africa (*4*), a knowledge gap exists in the ecology of AIV in tropical regions (*1**,**5*). Whether AIV circulate in waterbird communities in Africa and whether tropical ecosystems can play a role in the perpetuation of AIV among waterfowl remain unknown. We report results from large-scale surveillance of water birds in 12 countries in Africa (Figure).

**Figure:**
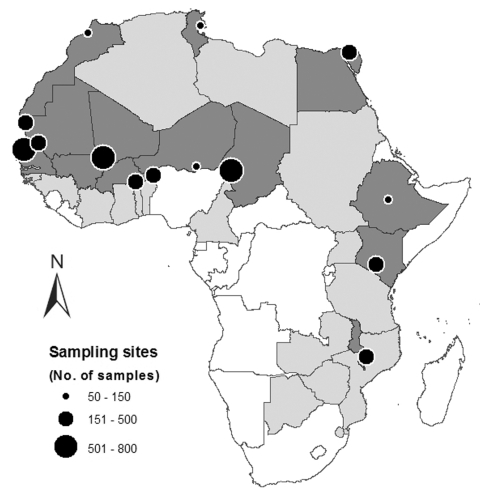
Locations of sampling sites (or clusters of sites) in surveyed African countries (dark gray) initially participating in the Food and Agriculture Organization’s Technical Cooperation Programs (light and dark gray). All samples were collected from mid-January to early March 2006 (but until May in Tunisia).

## The Study

This surveillance program was implemented in early 2006 within the framework of the Food and Agriculture Organization (FAO)’s Technical Cooperation Programs of Emergency Assistance for Early Detection and Prevention of Avian Influenza. Field sampling operations were coordinated by Centre de cooperation Internationale en Recherche Agronomique pour le Développement and by Wetlands International, in partnership with wildlife and veterinary national services, international organizations^1^), local ornithologic nongovernment organizations, as well as national hunting associations and safari operators. Study species were selected among bird families recognized as major AIV reservoirs (notably among the orders Anseriformes and Charadriiformes), in both Eurasian and Afro-tropical bird communities. Study sites important for congregatory water birds were selected in accordance with national surveillance programs and field logistic constraints and included sites where palearctic and Afro-tropical birds mix.

From mid-January to early March 2006 (and early May in Tunisia), we collected cloacal swab samples from captured birds and from freshly killed birds provided by hunters. Samples of fresh droppings were also collected at roosting areas for gulls, terns, and some ducks. In Ethiopia, which has hunting restrictions, and in countries in which emergency surveillance operations were implemented after notification of influenza A (H5N1) outbreaks in Nigeria (Burkina Faso, Niger), special permits were obtained to shoot birds for sample collection (n = 732).

Materials used and storing procedures were standardized among field teams. The transport medium consisted of an isotonic phosphate-buffered saline, pH 7.0–7.4, containing antimicrobial agents (penicillin 10,000 U/mL, streptomycin 10 mg/mL, amphotericin B 25 μg/mL, and gentamycin 250 μg/mL) supplemented with 10% glycerol. Samples were stored in liquid nitrogen containers or on ice and then stored at <–70°C after a few hours (generally <4 h, maximum of 24 h). They were shipped in dry ice in cryopacks until processed.

Samples were analyzed at the Istituto Zooprofilattico Sperimentale delle Venezie (Italy), except for samples from Egypt that were analyzed at the US Naval Medical Research Unit-3 (Egypt), samples from Kenya and Malawi which were analyzed at the Agricultural Research Council Onderstepoort Veterinary Institute (RSA), and samples from Tunisia which were analyzed at the Southeast Poultry Research Laboratory (USA). The samples were all screened by real-time reverse transcription (RT)–PCR specific for type A influenza viruses (*6*), and positive samples were tested by RT-PCR specific for H5 subtype. All type A–positive samples were subsequently processed for virus isolation by using standard methods (inoculation into the allantoic cavity of 9- to 10-day-old embryonated specific-pathogen–free eggs, EU directive 92/40). Isolates were characterized by hemagglutination and neuraminidase-inhibition tests by using specific hyperimmune chicken antisera to the reference strains of influenza virus (*7*). Molecular pathogenicity of H5 subtype–positive samples was determined by sequencing the hemagglutinin gene segment (BigDye Terminator v3.1 cycle sequencing kit, Applied Biosystems, Foster City, CA, USA).

A total of 4,553 birds (Table 1), consisting mostly of Afro-tropical and Eurasian ducks (32% and 31% of samples, respectively), were tested. The overall protion of AIV detected was 3.5% (n = 159 RT-PCR–positive samples, including both cloacal swabs and fresh droppings). Low-pathogenicity AIV were detected in 14 species of ducks, waders, gulls, terns, and rails, including both Eurasian and Afro-tropical species (Table 1). Positive samples were obtained from 8 countries (Chad, Ethiopia, Mali, Mauritania, Morocco, Niger, Senegal, and Tunisia). In the 2 most frequently sampled species, Eurasian ducks (garganey [*Anas querquedula*], n = 1,329) and Afro-tropical duck (white-faced whistling ducks [*Dendrocygna viduata*], n = 1,157), AIV were detected from most surveyed countries but with a highly variable prevalence (Table 2). Neither influenza A (H5N1) viruses nor any highly pathogenic AIV were detected. A total of 11 samples were positive for H5 subtype, mostly from garganey ducks (H5 prevalence of 0.7%). Finally, 5 low-pathogenicity AIV were isolated: 3 distinct isolates that originated from garganey ducks sampled in the Inner Niger Delta in Mali (H5N3, H11N9, H12N5) and 2 isolates that originated from white-faced whistling ducks sampled in Ethiopia (H8N4) and Senegal (H1N1).

**Table 1:** Prevalence of avian Influenza virus in wild birds*

Bird group	Species tested	No.	PCR positive, no. (%)	Positive country
African ducks	9 species (total, including 4 named below)	1,455	41 (2.8)	
	*Dendrocygna viduata*	1,181	38 (3.2)	TD, ET, ML, MR, NE, SN
	*Sarkidiornis melanotos*	117	3 (2.6)	ML, NE
	*D. bicolor*	88	0	
	*Plectropterus gambensis*	32	0	
Eurasian ducks	10 species	1,409	93 (6.6)	
	*Anas querquedula*	1,335	87 (6.5)	TD, ML, MR, NE, SN
	*A. acuta*	24	2 (8.3)	ML
	*A. crecca*	24	3 (12.5)	MA
	*A. clypeata*	6	1 (16.7)	MA
Eurasian waders	13 species	409	6 (1.5)	
	*Philomachus pugnax*	115	2 (1.7)	ML
	*Tringa glareola*	74	0	
	*Calidris minuta*	60	0	
	*C. ferruginea*	45	2 (4.4)	TN
	*Himantopus himantopus*	45	0	
	*Gallinago gallinago*	30	0	
	*T. erythropus*	23	2 (8.7)	ML
Rails	8 species	438	3 (0.7)	
	*Porphyrio alleni*	187	0	
	*Amaurornis flavirostris*	88	0	
	*Fulica cristata*	80	0	
	*Gallinula chloropus*	31	2 (6.5)	ML
	*Porphyrio porphyrio*	10	1 (10)	ML
Gulls	3 species	366	14 (3.8)	
	*Larus genei*	156	13 (8.3)	SN
	*L. fuscus*	129	1 (0.8)	MR
	*L. melanocephalus*	81	0	
Terns	7 species	159	2 (1.3)	
	*Sterna* sp.†	150	2 (1.3)	MR
Cormorants	2 species	148	0	
	*Phalacrocorax carbo*	130	0	
Other	36 species	196	0	
Total	87 species	4,553	159 (3.5)	

*Detected by reverse transcription–PCR (RT-PCR), for all RT-PCR–positive species and in species with >**30 individuals sampled.** Lower numbers in individual species are included in the total for each bird group. Countries where RT-PCR–positive samples were obtained are indicated (TD, Chad; ET, Ethiopia; ML, Mali; MR, Mauritania; NE, Niger; SN, Senegal; MA, Morocco; TN, Tunisia). †Unidentified fresh dropping samples from a multispecies flock of *Sterna caspia*, *S. maxima,* and *S. sandvicensis*.

**Table 2:** Reverse transcription PCR–based detection of influenza A virus in 2 wild duck species sampled in different countries

Species	Country	No. samples tested	No. PCR positive (%)
Garganey (*Anas querquedula*)	Chad	381	11 (2.9)
	Kenya	104	0
	Mali	411	22 (5.4)
	Mauritania	225	33 (14.7)
	Niger	87	4 (4.6)
	Senegal	121	17 (14.0)
White-faced whistling duck (*Dendrocygna viduata*)	Burkina Faso	167	0
	Chad	232	1 (0.4)
	Ethiopia	76	10 (13.2)
	Malawi	59	0
	Mali	36	1 (2.8)
	Mauritania	183	7 (3.8)
	Niger	232	8 (3.4)
	Senegal	172	11 (6.4)

## Conclusions

The African continent, in particular its sub-Saharan region, constitutes a seasonal shelter for a large number of Eurasian water birds, including an estimated 5.4 million ducks that gather in western and eastern Africa during the northern winter (*8*). In their overwintering sites, these birds congregate and mix with a wide variety of Afro-tropical water birds, some of them with large populations widespread over Africa.

AIV have been isolated in wild ducks on wintering grounds in both Europe and North America (*9**,**10*). Results from this surveillance program established that AIV are also present in wild birds in Africa during the northern winter. Low-pathogenicity AIV were detected and isolated in several species from several major wetlands of northern, western, and eastern Africa, which indicates that environmental conditions in Afro-tropical ecosystems are favorable for the persistence and transmission of AIV.

We detected and isolated AIV in Eurasian and Afro-tropical species. This finding shows that AIV circulate in migratory water birds originating from Eurasia and in African species that remain in the continent throughout the year. Moreover, the detection of viruses in some Eurasian wader species during wintering (in January in Mali) and during migration (in May in Tunisia) contrasts with the apparent absence of AIV reported from previous studies of waders in Europe (*5*,*11*). Since waders form the most abundant group of African-Eurasian migratory water birds (*12*), these shorebirds may play a role in maintaining some AIV in waterbird communities at wintering and stopover sites.

The detection of AIV in Eurasian ducks in several of their major overwintering sites in West Africa (e.g., the Inner Niger Delta, the Senegal River Delta, and Lake Chad) supports the hypothesis that AIV can persist in wild duck populations year-round through a continuous circulation in a proportion of birds (*1*). Variability in the prevalence observed might be related to differences in local logistical constrains but also to differences between African regions in their waterbird assemblage and connectivity with European breeding grounds. The different isolates obtained from garganey from the Inner Niger Delta also indicate that various subtypes are circulating at the same time in a population, a finding that agrees with patterns observed in Europe and North America (*11*,*13*).

Various AIV subtypes were isolated from apparently healthy garganey and white-faced whistling ducks, which indicates that both Eurasian and Afro-tropical ducks may serve as reservoirs of AIV. These results not only suggest that some Eurasian ducks could carry AIV on their northward spring migration but also raise the possibility that AIV could persist in the tropical region and be disseminated over Africa through intra-African migratory ducks. The presence of AIV at African wintering and stopover sites, where birds from various geographic origins congregate and mix, provides opportunities for transmission of AIV between different populations and spread of AIV over extensive areas in both Eurasia and Africa.

## Footnotes

1 African Waterbird Ringing Scheme (AFRING), Oiseaux Migrateurs du Paléarctique Occidental (OMPO), Office National de la Chasse et de la Faune Sauvage (ONCFS) , Dutch Centre for Field Ornithology or Stichting Openbaar Voortgezet Onderwijs Noord (SOVON), and Wildfowl and Wetlands Trust (WWT).
